# Genetic predisposition to ductal carcinoma *in situ* of the breast

**DOI:** 10.1186/s13058-016-0675-7

**Published:** 2016-02-17

**Authors:** Christos Petridis, Mark N. Brook, Vandna Shah, Kelly Kohut, Patricia Gorman, Michele Caneppele, Dina Levi, Efterpi Papouli, Nick Orr, Angela Cox, Simon S. Cross, Isabel dos-Santos-Silva, Julian Peto, Anthony Swerdlow, Minouk J. Schoemaker, Manjeet K. Bolla, Qin Wang, Joe Dennis, Kyriaki Michailidou, Javier Benitez, Anna González-Neira, Daniel C. Tessier, Daniel Vincent, Jingmei Li, Jonine Figueroa, Vessela Kristensen, Anne-Lise Borresen-Dale, Penny Soucy, Jacques Simard, Roger L. Milne, Graham G. Giles, Sara Margolin, Annika Lindblom, Thomas Brüning, Hiltrud Brauch, Melissa C. Southey, John L. Hopper, Thilo Dörk, Natalia V. Bogdanova, Maria Kabisch, Ute Hamann, Rita K. Schmutzler, Alfons Meindl, Hermann Brenner, Volker Arndt, Robert Winqvist, Katri Pylkäs, Peter A. Fasching, Matthias W. Beckmann, Jan Lubinski, Anna Jakubowska, Anna Marie Mulligan, Irene L. Andrulis, Rob A. E. M. Tollenaar, Peter Devilee, Loic Le Marchand, Christopher A. Haiman, Arto Mannermaa, Veli-Matti Kosma, Paolo Radice, Paolo Peterlongo, Frederik Marme, Barbara Burwinkel, Carolien H. M. van Deurzen, Antoinette Hollestelle, Nicola Miller, Michael J. Kerin, Diether Lambrechts, Giuseppe Floris, Jelle Wesseling, Henrik Flyger, Stig E. Bojesen, Song Yao, Christine B. Ambrosone, Georgia Chenevix-Trench, Thérèse Truong, Pascal Guénel, Anja Rudolph, Jenny Chang-Claude, Heli Nevanlinna, Carl Blomqvist, Kamila Czene, Judith S. Brand, Janet E. Olson, Fergus J. Couch, Alison M. Dunning, Per Hall, Douglas F. Easton, Paul D. P. Pharoah, Sarah E. Pinder, Marjanka K Schmidt, Ian Tomlinson, Rebecca Roylance, Montserrat García-Closas, Elinor J. Sawyer

**Affiliations:** Research Oncology, Guy’s Hospital, King’s College London, London, UK; Medical and Molecular Genetics, Guy’s Hospital, King’s College London, London, UK; Division of Genetics and Epidemiology, The Institute of Cancer Research, London, UK; Centre for Molecular Oncology, Barts Cancer Institute, Queen Mary University of London, London, UK; Biomedical Research Centre, King’s College London, Guy’s Hospital, London, UK; The Breast Cancer Now Toby Robins Research Centre, The Institute of Cancer Research, London, UK; Sheffield Cancer Research, Department of Oncology, University of Sheffield, Sheffield, UK; Academic Unit of Pathology, Department of Neuroscience, University of Sheffield, Sheffield, UK; Department of Non-Communicable Disease Epidemiology, London School of Hygiene and Tropical Medicine, London, UK; Division of Breast Cancer Research, The Institute of Cancer Research, London, UK; Centre for Cancer Genetic Epidemiology, Department of Public Health and Primary Care, University of Cambridge, Cambridge, UK; Human Cancer Genetics Program, Spanish National Cancer Research Centre, Madrid, Spain; Centro de Investigación en Red de Enfermedades Raras, Valencia, Spain; Centre d’innovation Génome Québec et Université McGill, Montréal, Canada; Department of Medical Epidemiology and Biostatistics, Karolinska Institutet, Stockholm, Sweden; Division of Cancer Epidemiology and Genetics, National Cancer Institute, Rockville, MD USA; Department of Genetics, Institute for Cancer Research, Oslo University Hospital Radiumhospitalet, Oslo, Norway; K.G. Jebsen Center for Breast Cancer Research, Institute of Clinical Medicine, Faculty of Medicine, University of Oslo, Oslo, Norway; Department of Clinical Molecular Biology, Oslo University Hospital, University of Oslo, Oslo, Norway; Genomics Center, Centre Hospitalier Universitaire de Québec Research Center, Laval University, Québec City, Canada; Cancer Epidemiology Centre, Cancer Council Victoria, Melbourne, Australia; Centre for Epidemiology and Biostatistics, Melbourne School of Population and Global health, The University of Melbourne, Melbourne, VIC Australia; Department of Oncology - Pathology, Karolinska Institutet, Stockholm, Sweden; Department of Molecular Medicine and Surgery, Karolinska Institutet, Stockholm, Sweden; Institute for Prevention and Occupational Medicine of the German Social Accident Insurance, Institute of the Ruhr University Bochum, Bochum, Germany; Dr. Margarete Fischer-Bosch-Institute of Clinical Pharmacology, Stuttgart, Germany; University of Tübingen, Tübingen, Germany; German Cancer Consortium (DKTK), German Cancer Research Center (DKFZ), Heidelberg, Germany; Department of Pathology, The University of Melbourne, Melbourne, Australia; Gynaecology Research Unit, Hannover Medical School, Hannover, Germany; Department of Radiation Oncology, Hannover Medical School, Hannover, Germany; Molecular Genetics of Breast Cancer, German Cancer Research Center (DKFZ), Heidelberg, Germany; Center for Familial Breast and Ovarian Cancer, Medical Faculty, University of Cologne and University Hospital Cologne, Cologne, Germany; Center for Integrated Oncology (CIO), Medical Faculty, University of Cologne and University Hospital Cologne, Cologne, Germany; Center for Molecular Medicine Cologne (CMMC), Medical Faculty, University of Cologne and University Hospital Cologne, Cologne, Germany; Division of Gynaecology and Obstetrics, Technische Universität München, Munich, Germany; Division of Clinical Epidemiology and Aging Research, German Cancer Research Center (DKFZ), Heidelberg, Germany; Division of Preventive Oncology, German Cancer Research Center (DKFZ), Heidelberg, Germany; Laboratory of Cancer Genetics and Tumor Biology, Cancer and Translational Medicine Research Unit, Biocenter Oulu, University of Oulu, Oulu, Finland; Laboratory of Cancer Genetics and Tumor Biology, Northern Finland Laboratory Centre NordLab, Oulu, Finland; Department of Gynaecology and Obstetrics, University Hospital Erlangen, Friedrich-Alexander University Erlangen-Nuremberg, Comprehensive Cancer Center Erlangen-EMN, Erlangen, Germany; David Geffen School of Medicine, Department of Medicine Division of Hematology and Oncology, University of California at Los Angeles, Los Angeles, CA USA; Department of Genetics and Pathology, Pomeranian Medical University, Szczecin, Poland; Department of Laboratory Medicine and Pathobiology, University of Toronto, Toronto, Canada; Laboratory Medicine Program, University Health Network, Toronto, Canada; Lunenfeld-Tanenbaum Research Institute of Mount Sinai Hospital, Toronto, Canada; Department of Molecular Genetics, University of Toronto, Toronto, Canada; Department of Surgery, Leiden University Medical Center, Leiden, The Netherlands; Department of Pathology, Leiden University Medical Center, Leiden, The Netherlands; Department of Human Genetics, Leiden University Medical Center, Leiden, The Netherlands; University of Hawaii Cancer Center, Honolulu, HI USA; Department of Preventive Medicine, Keck School of Medicine, University of Southern California, Los Angeles, CA USA; Imaging Center, Department of Clinical Pathology, Kuopio University Hospital, Kuopio, Finland; Institute of Clinical Medicine, Pathology and Forensic Medicine, University of Eastern Finland, Kuopio, Finland; Cancer Center of Eastern Finland, University of Eastern Finland, Kuopio, Finland; Unit of Molecular Bases of Genetic Risk and Genetic Testing, Department of Preventive and Predictive Medicine, Fondazione IRCCS (Istituto Di Ricovero e Cura a Carattere Scientifico) Istituto Nazionale dei Tumori (INT), Milan, Italy; IFOM, Fondazione Istituto FIRC (Italian Foundation of Cancer Research) di Oncologia Molecolare, Milan, Italy; National Center for Tumor Diseases, University of Heidelberg, Heidelberg, Germany; Department of Obstetrics and Gynecology, University of Heidelberg, Heidelberg, Germany; Molecular Epidemiology Group, German Cancer Research Center (DKFZ), Heidelberg, Germany; Department of Pathology, Erasmus University Medical Center, Rotterdam, The Netherlands; Department of Medical Oncology, Family Cancer Clinic, Erasmus MC Cancer Institute, Rotterdam, The Netherlands; School of Medicine, National University of Ireland, Galway, Ireland; Vesalius Research Center, VIB, Leuven, Belgium; Laboratory for Translational Genetics, Department of Oncology, University of Leuven, Leuven, Belgium; University Hospital Gashuisberg, Leuven, Belgium; Netherlands Cancer Institute, Antoni van Leeuwenhoek hospital, Amsterdam, The Netherlands; Department of Breast Surgery, Herlev Hospital, Copenhagen University Hospital, Herlev, Denmark; Copenhagen General Population Study, Herlev Hospital, Copenhagen University Hospital, Herlev, Denmark; Department of Clinical Biochemistry, Herlev Hospital, Copenhagen University Hospital, Herlev, Denmark; Faculty of Health and Medical Sciences, University of Copenhagen, Copenhagen, Denmark; Department of Cancer Prevention and Control, Roswell Park Cancer Institute, Buffalo, NY USA; Roswell Park Cancer Institute, Buffalo, NY USA; Department of Genetics, QIMR Berghofer Medical Research Institute, Brisbane, Australia; Environmental Epidemiology of Cancer, Center for Research in Epidemiology and Population Health, INSERM, Villejuif, France; University Paris-Sud, Villejuif, France; Division of Cancer Epidemiology, German Cancer Research Center (DKFZ), Heidelberg, Germany; Department of Obstetrics and Gynecology, Helsinki University Hospital, University of Helsinki, Helsinki, Finland; Department of Oncology, Helsinki University Hospital, University of Helsinki, Helsinki, Finland; Department of Health Sciences Research, Mayo Clinic, Rochester, MN USA; Department of Laboratory Medicine and Pathology, Mayo Clinic, Rochester, MN USA; Centre for Cancer Genetic Epidemiology, Department of Oncology, University of Cambridge, Cambridge, UK; Wellcome Trust Centre for Human Genetics and Oxford NIHR Biomedical Research Centre, University of Oxford, Oxford, UK

**Keywords:** Ductal carcinoma in situ, Association study, Genetic predisposition, Common variants

## Abstract

**Background:**

Ductal carcinoma *in situ* (DCIS) is a non-invasive form of breast cancer. It is often associated with invasive ductal carcinoma (IDC), and is considered to be a non-obligate precursor of IDC. It is not clear to what extent these two forms of cancer share low-risk susceptibility loci, or whether there are differences in the strength of association for shared loci.

**Methods:**

To identify genetic polymorphisms that predispose to DCIS, we pooled data from 38 studies comprising 5,067 cases of DCIS, 24,584 cases of IDC and 37,467 controls, all genotyped using the iCOGS chip.

**Results:**

Most (67 %) of the 76 known breast cancer predisposition loci showed an association with DCIS in the same direction as previously reported for invasive breast cancer. Case-only analysis showed no evidence for differences between associations for IDC and DCIS after considering multiple testing.

Analysis by estrogen receptor (ER) status confirmed that loci associated with ER positive IDC were also associated with ER positive DCIS. Analysis of DCIS by grade suggested that two independent SNPs at 11q13.3 near *CCND1* were specific to low/intermediate grade DCIS (rs75915166, rs554219). These associations with grade remained after adjusting for ER status and were also found in IDC.

We found no novel DCIS-specific loci at a genome wide significance level of *P* < 5.0x10^-8^.

**Conclusion:**

In conclusion, this study provides the strongest evidence to date of a shared genetic susceptibility for IDC and DCIS. Studies with larger numbers of DCIS are needed to determine if IDC or DCIS specific loci exist.

**Electronic supplementary material:**

The online version of this article (doi:10.1186/s13058-016-0675-7) contains supplementary material, which is available to authorized users.

## Background

Ductal carcinoma in situ (DCIS) is a non-obligate precursor of invasive breast cancer including invasive ductal/no special type carcinomas (IDC). Since the introduction of screening mammography there has been a 7-fold increase in reported DCIS incidence in the USA, primarily in postmenopausal women [1], with about 20 % of screen-detected tumors being DCIS [2]. Approximately 45–78 % of all invasive breast cancers are associated with DCIS [3, 4]. It is hypothesized in the majority of these cases that the invasive component has arisen from the DCIS as they generally share the same somatic genetic changes. The proportion of IDC associated with DCIS varies depending on subtype, with luminal and human epidermal growth factor receptor 2 (HER2)-positive IDC having more frequent DCIS (53 % and 63 %, respectively) than invasive basal breast cancers (33 %) [5].

As most DCIS is treated surgically, the natural progression of untreated DCIS is not known. However, in one small study of patients with predominantly low-grade DCIS misdiagnosed as benign breast disease and who received no surgical intervention, 6 out of 13 patients developed ipsilateral invasive carcinoma with mean time to the development of invasive carcinoma being 9.0 years [6]. In two specific DCIS trials in which DCIS was treated with breast-conserving surgery alone with no radiotherapy, long-term follow up shows that up to 30 % of women develop a recurrence (half of which will be DCIS and half invasive cancer) by 10 years [7].

Methods for accurately predicting the behavior of DCIS are poor [8]. Although grade has not been shown to be a good predictor of recurrence many clinicians use this classification to determine the use of radiotherapy following breast-conserving surgery. There is a strong correlation between the grade of the *in situ* and co-existing invasive components in IDC, suggesting that DCIS does not progress from low through to high grade before becoming invasive [9, 10].

Most non-genetic risk factors for breast cancer have similar associations with DCIS and IDC, supporting the notion that DCIS is a precursor of invasive cancer [11, 12]. There is also evidence from epidemiological studies that there is an inherited predisposition to DCIS. Women with DCIS have been shown to be 2.4 times (95 % CI 0.8, 7.2) more likely to have an affected mother and sister with breast cancer than controls [13]. Furthermore, there is evidence from a study of almost 40,000 women that the familial relative risk of DCIS is greater than that of invasive breast cancer. For women aged 30–49 years with a family history of breast cancer the odds ratio (OR) for developing DCIS was calculated as 2.4 (95 % CI 1.1, 4.9) compared to 1.7 (95 % CI 0.9, 3.4) for invasive cancer. For women aged 50 years and above the risks were slightly reduced, but still higher for DCIS (OR = 2.2, 95 % CI 1.0, 4.2) than invasive disease (OR = 1.5, 95 % CI 1.0, 2.2) [14]. However, this was not confirmed in the Million Women Study, in which the association with family history was similar for DCIS and IDC [12].

A small part of this inherited predisposition is explained by *BRCA1/2* mutations, as mutations in these genes are found in a similar proportion of DCIS and invasive breast cancer cases [15]. For low-risk common breast cancer predisposition alleles most of the initial breast cancer association studies have not been powered to identify associations with DCIS, so it is not clear whether all the low-risk susceptibility loci that have been identified are associated with DCIS and what the strength of any associations are.

It is now evident that some low-risk susceptibility loci are associated with different pathological subtypes of breast cancer and support the hypothesis that breast tumor subtypes arise through distinct molecular pathways [16–18]. In order to identify further low-risk susceptibility loci, it will be necessary to look at specific morphological subtypes including DCIS and the cytonuclear grade and estrogen receptor (ER) status of the disease. In this study we analyzed 3,078 cases of pure DCIS collected through the ICICLE study (a study to Investigate the genetics of In situ Carcinoma of the ductaL subtype) and performed a meta-analysis with 2,352 *in situ* cases collected through the Breast Cancer Association Consortium (BCAC). Our aims were to assess whether any of the known low-risk breast susceptibility alleles have different associations for DCIS and IDC, and to identify if there are any DCIS-specific low-risk alleles.

## Methods

### Ethics statement

All studies were performed with ethical committee approval (listed in acknowledgements) and subjects participated in the studies after providing informed consent.

### Study populations

Cases came from ICICLE (MREC 08/H0502/4), a UK study of DCIS, and from 37 studies forming part of the BCAC included in the Collaborative Oncological Gene-Environment Study (COGS) [19] (Additional file 1). The ICICLE study recruited patients from participating centers throughout the UK with the aim of identifying predisposition genes for DCIS. Patients aged 60 years or less at the time of diagnosis, with a current or past history of DCIS (without invasive disease of any histological subtype) were eligible. A total of 3,078 subjects were recruited following identification from local pathology reports in 97 UK hospitals. All cases were genotyped with the iCOGS chip and compared to 5,000 UK controls selected from four UK studies (BBCS 1,231 controls, SBCS 704 controls, UKBGS 370 controls, SEARCH 2,695 controls) participating in BCAC (Additional file 2) and already typed on the iCOGS chip. Controls were randomly selected prior to analysis, and were excluded from case–control comparisons with BCAC cases from the originating study. After excluding individuals based on genotyping quality (see subsection “Genotyping and analysis”) and non-European ancestry, data for the ICICLE study available for analysis included 2,715 subjects with DCIS (cases) and 4,813 controls.

Women with all types of breast cancer were recruited into the BCAC studies. Pathological information in BCAC was collected in the individual studies but was also combined and checked through standardized data control in a central database. A total of 2,352 subjects with DCIS were identified in the central BCAC pathology database (see Additional file 3 for number of cases by study). Controls came from the 37 BCAC studies (37,654 in total).

### Genotyping and analysis

After DNA extraction from peripheral blood, ICICLE samples were genotyped on the iCOGS custom Illumina iSelect array (Illumina, San Diego, CA), which contains 211,155 single nucleotide polymorphisms (SNPs), at King’s College London. The remaining cases and controls were genotyped as part of the COGS project described in detail elsewhere [19]. The ICICLE cases were analyzed using the same quality control (QC) criteria as the COGS project. Briefly, genotypes were called using Illumina’s proprietary GenCall algorithm and 10,000 SNPs were manually inspected to verify the algorithm calls. Individuals were excluded if genotypically non-European or not female, or had an overall call rate <95 %. SNPs were excluded with a Gen-Train score <0.4, call rate <95 % (call rate <99 % if minor allele frequency (MAF) was <0.1) and Hardy Weinberg equilibrium (HWE) value of *P* <10^-7^ or evidence of poor clustering on inspection of cluster plots. All SNPs with MAF <0.01 were excluded. A cryptic relatedness analysis of the whole dataset was performed using 46,789 uncorrelated SNPs and led to the exclusion of 28 cases and 18 controls due to relatedness between the ICICLE and BCAC samples (PIHAT >0.1875).

For ICICLE cases and controls, principal component analysis (PCA) was carried out on a subset of 46,789 uncorrelated SNPs and individuals or groups distinct from the main cluster (327 cases and 164 controls) were excluded using the first five principal components (PCs) (Additional file 4). Following removal of outliers, the PCA was repeated and the first five PCs were included as covariates in the analysis.

The adequacy of the case–control matching was evaluated using quantile-quantile plots of test statistics and the inflation factor (*λ*) calculated using 37,289 uncorrelated SNPs that were not selected by BCAC and were not within one of the four common fine-mapping regions, to minimize selection for SNPs associated with breast cancer (Additional file 5). As the majority of the SNPs on the iCOGS array are associated with breast, ovarian or prostate cancer, the SNPs selected for this analysis were taken from the set of prostate cancer SNPs, with the assumption that these SNPs were more likely to be representative of common SNPs in terms of population structure in our study.

For each SNP, we estimated a per-allele OR and reported corresponding 95 % CI using logistic regression analysis, including the five PCs as covariates, using PLINK v1.07 (http://pngu.mgh.harvard.edu/~purcell/plink/).

Genotyping and analysis of BCAC studies have been described in detail elsewhere [19]. In brief, data were analyzed using the Genotype Library and Utilities (GLU) package to estimate per-allele ORs for each SNP using unconditional logistic regression. All analyses were performed in subjects of European ancestry (determined by PC analyses) and adjusted for study and seven principal components.

Case–control ORs for DCIS cases vs controls from BCAC and ICICLE were combined using inverse variance-weighted fixed-effects meta-analysis, as implemented in METAL [20]. Case-only analyses were also carried out to compare genotype frequencies for (1) ER-positive (ER+) vs ER-negative (ER–) DCIS, (2) high grade DCIS vs low and intermediate grade DCIS, and (3) DCIS vs IDC (see Additional file 3 for number of cases by study), (4) DCIS diagnosis in patients <50 years of age vs DCIS diagnosis in patients ≥50 years, and were used as a test for heterogeneity of ORs by tumor subtype/age (see Additional file 6 for number of cases by group). Only studies with data on both subtypes contributed to case-only analysis comparing these subtypes. Similar case-only analyses were performed for the IDC cases in these studies to assess whether any heterogeneity evident in DCIS also occurred in IDC.

Novel SNPs showing the strongest evidence of association with DCIS (*P* <6 × 10^-6^) in the meta-analysis (after excluding previously reported loci) were genotyped in a phase II analysis at LGC Genomics (LGC, Teddington, UK). The phase II samples consisted of 653 DCIS cases from the ICICLE and Breakthrough Generation Studies and 1,882 controls from the ICICLE study not previously genotyped on the iCOGS chip. All individuals included in the analysis were of European ancestry (self-reported).

For the known breast cancer predisposition loci *P* <0.00066 was considered statistically significant (with Bonferroni correction for multiple testing on 76 known loci). All of the known breast cancer susceptibility loci were included in the iCOGS chip with the exception of rs2284378 (20q11), which was identified as an ER– breast cancer predisposition SNP after the iCOGS chip was developed [21].

### Assessment of grade and ER status

For the ICICLE study, information on cytonuclear grade of DCIS was available for 2,578 cases, mostly from the local histopathology reports. In 200 cases where the grade data were missing from the report but the tumor block was available, an H&E section was cut and the DCIS was graded by the study histopathologist (SEP) according to UK and College of American Pathologists guidelines [22]. Data on grade of DCIS were available from histopathology reports for 828 BCAC cases.

A subset of 81 ICICLE cases, graded in the pathology report and with a tumor block available, were examined to assess the reliability of the cytonuclear grade provided by the pathology reports. In the majority of cases (86.5 %) grade was concordant with the pathology report. Nine cases were re-graded as low/intermediate grade and two cases as high grade. As the study pathologist re-graded the samples on a single H&E section, rather than all the blocks from an individual case, and in some cases on re-excision specimens with residual disease rather than the original excision specimen, the grade reported in the pathology report, if available, was used for the purposes of this study.

ER status from local histopathology reports was available for 1,086 ICICLE cases. For the remaining 781 ICICLE cases where the tumor block was available, immunohistochemistry was performed on 3-μM sections, which were incubated at 60 °C for 1 h prior to automated staining using the VENTANA®. Estrogen receptor staining was carried out using CONFIRM™ anti-estrogen receptor (SP1) rabbit monoclonal primary antibody (Catalog number 790-4324) with no variation to the recommended protocol. ER staining was scored by three independent reviewers (CP, VS, DLe) using the Allred method, and any discrepancies were reviewed by the study histopathologist (SEP). DCIS with an Allred score ≥3 was considered ER+ and DCIS with scores of 0–2 (approximately equivalent to <1 % of nuclei) was regarded as ER–. ER status was available on 965 cases from BCAC (Additional file 6).

## Results

### Assessment of known breast cancer susceptibility loci for association with DCIS

For the majority of known loci (n = 46) the risk allele for invasive breast cancer is the minor allele. For the ORs presented here the reference allele was set as the non-risk allele to make it clear whether the association with DCIS was in the same direction as previously published for invasive breast cancer. Thus, ORs for DCIS will be >1 if in the same direction as invasive disease and <1 if in the opposite direction.

**Fig. 1:**
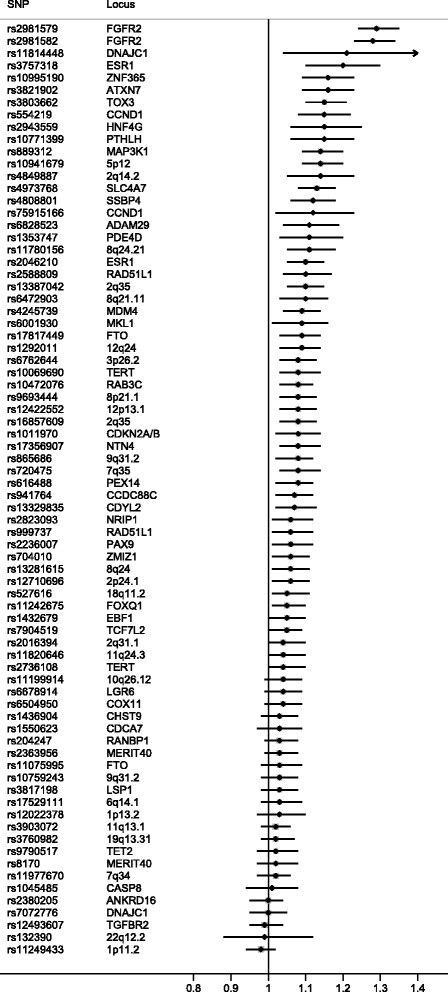
Known breast cancer predisposition loci for ductal carcinoma *in situ* plotted according to the risk allele for invasive disease. Odds ratios >1 indicate that the association is in the same direction as previously published for invasive breast cancer

Of the 76 known common breast cancer susceptibility loci genotyped on the iCOGS array, 51 were associated with DCIS (*P* <0.05), with the effect in the same direction as previously reported in IDC (Fig. 1 and Additional file 7). Sixteen SNPs were significantly associated with DCIS (*P* <0.00066) with three being genome-wide significant (*P* <5 × 10^-8^, Table 1). The strongest associations were with for loci in *FGFR2* (rs2981579: OR 1.29, 95 % CI 1.24, 1.35; *P* = 9.0 × 10^-30^) and *TOX3* (rs3803662: OR 1.15, 95 % CI 1.1, 1.21; *P* = 1.7 × 10^-8^).

**Table 1:** Loci showing a significant association with ductal carcinoma *in situ* (DCIS) at *P* <0.00066

Chromosome	SNP	Locus	RAF	DCIS vs controls (meta-analysis)			IDC vs controls			Case-only DCIS vs IDC
			Controls	OR	(95 % CI)	*P*	OR	(95 % CI)	*P*	*P*-Het
10	rs2981579	FGFR2	0.40	1.29	(1.24, 1.35)	9.0 × 10^-30^	1.24	(1.21, 1.28)	6.1 × 10^-66^	0.14
10	rs2981582	FGFR2	0.38	1.28	(1.23, 1.34)	1.8 × 10^-27^	1.23	(1.20, 1.26)	2.1 × 10^-59^	0.21
16	rs3803662	TOX3	0.26	1.15	(1.10, 1.21)	1.7 × 10^-8^	1.23	(1.20, 1.27)	1.5 × 10^-50^	0.69
5	rs889312	MAP3K1	0.28	1.14	(1.09, 1.20)	6.9 × 10^-8^	1.11	(1.08, 1.14)	2.2 × 10^-14^	0.13
3	rs4973768	SLC4A7	0.47	1.13	(1.08, 1.18)	9.1 × 10^-8^	1.09	(1.07, 1.12)	8.2 × 10^-13^	0.58
5	rs10941679	5p12	0.25	1.14	(1.09, 1.20)	1.3 × 10^-7^	1.14	(1.11, 1.18)	1.2 × 10^-20^	0.90
3	rs3821902	ATXN7	0.13	1.16	(1.09, 1.23)	3.0 × 10^-6^	1.06	(1.02, 1.09)	0.0030	0.33
19	rs4808801	SSBP4	0.65	1.12	(1.06, 1.18)	3.1 × 10^-6^	1.09	(1.05, 1.11)	3.5 × 10^-9^	0.16
10	rs10995190	ZNF365	0.85	1.16	(1.09, 1.23)	4.1 × 10^-6^	1.15	(1.11, 1.19)	7.5 × 10^-16^	0.61
2	rs13387042	2q35	0.51	1.10	(1.05, 1.15)	1.1 × 10^-5^	1.14	(1.11, 1.16)	8.3 × 10^-25^	0.34
6	rs3757318	ESR1	0.07	1.20	(1.10, 1.30)	1.4 × 10^-5^	1.16	(1.10, 1.21)	1.2 × 10^-9^	0.85
11	rs554219	CCND1	0.12	1.15	(1.08, 1.22)	2.8 × 10^-5^	1.27	(1.22, 1.32)	6.4 × 10^-38^	0.88
6	rs2046210	ESR1	0.34	1.10	(1.05, 1.15)	8.6 × 10^-5^	1.09	(1.06, 1.12)	4.0 × 10^-10^	0.32
12	rs10771399	PTHLH	0.88	1.15	(1.06, 1.23)	0.00021	1.18	(1.12, 1.22)	1.2 × 10^-14^	0.53
8	rs11780156	8q24.21	0.16	1.11	(1.05, 1.18)	0.00027	1.10	(1.06, 1.14)	2.3 × 10^-8^	0.88
16	rs17817449	FTO	0.60	1.09	(1.03, 1.14)	0.00052	1.06	(1.04, 1.10)	5.9 × 10^-7^	0.32

*SNP* single nucleotide polymorphism, *IDC* invasive ductal carcinoma, *OR* odds ratio; *P-He*t *P* value for heterogeneity; *RAF* risk allele frequency

The case-only analysis (DCIS vs IDC) confirmed the shared genetic susceptibility between DCIS and IDC as none of the heterogeneity *P* values (*P*-Het) were significant after Bonferroni adjustment for 76 SNPs (Additional file 7). The case-only analysis (DCIS diagnosed at <50 years vs ≥50 years of age) revealed one SNP (rs527616, 18q11.2) that was significantly associated with DCIS in younger women (*P*-Het_<50/≥50_ = 0.0003) even though the overall *P* value for DCIS was not statistically significant after Bonferroni correction (OR 1.05, 95 % CI 1.01, 1.11; *P* = 0.020) (Additional file 8).

### Assessment of known breast cancer susceptibility loci for association with DCIS by ER status

**Fig. 2:**
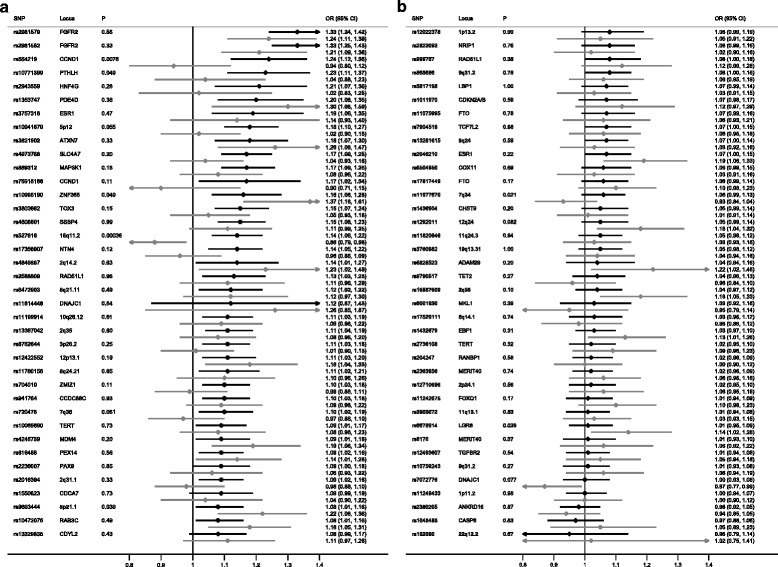
Known breast cancer predisposition loci for estrogen receptor-positive (ER+) (*black lines*) and ER– ductal carcinoma in situ (*gray lines*). Due to the large number of single nucleotide polymorphisms (*SNPs*), for better visual representation the plot is split into two different sections (**a** and **b**) with a descending order of effect size for the ER+ group. *OR* odds ratio

Following immunohistochemistry for ER in the ICICLE study samples, 1,484 cases (54 %) were classified as ER+ and 383 (14 %) as ER–. The ER data on BCAC DCIS were less complete with 664 (28 %) ER+, 301 (13 %) ER– and 1,387 cases (59 %) of unknown ER status (Additional file 6). Analysis by ER status confirmed that loci associated with ER+ IDC were also associated with ER+ DCIS (Fig. 2 and Additional file 9). These similarities were less clear for ER– DCIS and ER– IDC but this may be due to small numbers of ER– DCIS cases. A case-only analysis of ER+ vs ER– DCIS was not performed due to the small numbers of ER– cases.

### Assessment of known breast cancer susceptibility loci for association with DCIS by grade

Grade data were available for 95 % of ICICLE DCIS cases; 1,635 (60 %) were of high cytonuclear grade and 943 (35 %) of low/intermediate grade. The grade data on the BCAC DCIS were less complete with data only available for 35 % of cases: 306 (13 %) high grade and 522 (22 %) low/intermediate grade cases (Additional file 6). Case–control analysis was performed separately on the low/intermediate and high grade subsets and a case-only analysis of low/intermediate grade vs high grade DCIS was performed to assess whether any of these loci were grade-specific.

Analysis of DCIS by grade revealed that although the majority of SNPs predispose to all grades of DCIS, some are grade-specific (Additional files 10 and 11). The two SNPs close to *CCND1* were strongly associated with low/intermediate grade DCIS (rs75915166, OR 1.36, 95 % CI 1.17, 1.59; *P* = 7.2 × 10^-5^; rs554219, OR 1.32, 95 % CI 1.18, 1.48; *P* = 8.2 × 10^-7^) and there was no association with high grade DCIS (Table 2). Case-only analysis confirmed that these loci were low/intermediate grade-specific (rs75915166, *P*-Het_low/highgrade_ = 0.00014; rs554219, *P*-Het_low/highgrade_ = 0.00013) and this was independent of ER status (adjusted for ER status rs75915166, *P* = 0.0050; rs554219, *P* = 0.019).

**Table 2:** Association between rs75915166 or rs554219 and grade in ductal carcinoma *in situ*

		Meta-analysis				
		OR (95 % CI)	*P*	Low/intermediate grade, number	High grade, number	Controls, number
rs75915166						
Low/intermediate grade vs controls		1.36 (1.17, 1.59)	7.2 × 10^-5^	1,465		35,521
High grade vs controls		0.92 (0.79, 1.08)	0.31		1,941	32,202
Case-only high vs low/intermediate grade						
	Unadjusted	0.68 (0.55, 0.83)	1.4 × 10^-4^	1,307	1,941	
	unadjusted (only cases with ER status)	0.65 (0.51, 0.84)	1.1 × 10^-3^	791	1,360	
	adjusted for ER status	0.68 (0.52, 0.89)	0.0050	791	1,360	
	ER+ only	0.68 (0.55, 0.84)	5 × 10^-4^	709	985	
rs554219						
Low/intermediate grade vs controls		1.32 (1.18, 1.48)	8.2 × 10^-7^	1,465		35,521
High grade vs controls		1.02 (0.91, 1.14)	0.75		1,941	32,202
Case-only high vs low/intermediate grade						
	Unadjusted	0.75 (0.65, 0.87)	1.3 × 10^-4^	1,307	1,941	
	unadjusted (only cases with ER status)	0.75 (0.63, 0.88)	2.1 × 10^-4^	791	1,360	
	adjusted for ER status	0.80 (0.67, 0.96)	0.019	792	1,360	
	ER+ only	0.76 (0.65, 0.89)	6.7 × 10^-4^	709	985	

*OR* odds ratio, *ER* estrogen receptor

A similar-case-only analysis of IDC by grade confirmed that the two SNPs on 11q13.3 close to *CCND1* were also invasive grade 1/2-specific in IDC (rs75915166, OR 1.42, *P* = 1.7 × 10^-30^, *P*-Het = 2.8 × 10^-10^; rs554219, OR 1.39, *P* = 4.7 × 10^-49^, *P*-Het = 1.3 × 10^-17^) and again were independent of ER status (*P* = 1.3 × 10^-6^, *P* = 1.6 × 10^-6^, respectively) (Additional file 12). In addition, other grade-specific loci were identified including three (rs2363956, rs8170 and rs10069690) specific to grade 3 invasive disease (Additional file 13).

rs10941679, 5p12 were borderline associated with low/intermediate grade DCIS (OR 1.26, *P* = 2.1 × 10^-7^, *P*-Het_low/highgrade_ = 0.0033). This locus has previously been shown to be associated with low grade progesterone receptor (PR) + IDC [23]. There was no evidence of any high grade DCIS specific loci (Additional file 11).

### Search for new DCIS predisposition loci

All SNPs that were genome-wide significant (*P* <5 × 10^-8^) in the meta-analysis were correlated with one of the known breast cancer predisposition loci. There were three SNPs that were not correlated with known loci at *P* <6 × 10^-6^ (Table 3), all with very little evidence of an association with IDC.

**Table 3:** Potential new ductal carcinoma *in situ* susceptibility loci

Single nucleotide polymorphism	rs12631593	rs13236351	rs73179023
Chromosome	3	7	22
Position	60701884	97772513	43424477
Locus	FHIT	LMTK2	PACSIN2:TTLL1
Minor allele frequency	0.11	0.032	0.13
ICICLE DCIS phase I			
Odds ratio (95 % CI)	1.15 (1.04, 1.28)	1.31 (1.10, 1.56)	0.83 (0.75, 0.91)
*P*	0.0088	0.0029	0.00020
BCAC DCIS			
Odds ratio (95 % CI)	1.25 (1.14, 1.36)	1.3 (1.12, 1.51)	0.86 (0.79, 0.94)
*P*	1.0 × 10^-6^	0.00060	0.0012
Meta-analysis phase I			
Odds ratio (95 % CI)	1.21 (1.13, 1.29)	1.3 (1.16, 1.46)	0.85 (0.79, 0.90)
*P*	5.5 × 10^-8^	5.7 × 10^-6^	1.1 × 10^-6^
Phase II DCIS			
Odds ratio (95 % CI)	0.93 (0.76, 1.14)	0.91 (0.63, 1.31)	0.95 (0.78, 1.15)
*P*	0.49	0.61	0.57
Meta-analysis phase II			
Odds ratio (95 % CI)	1.18 (1.10, 1.25)	1.26 (1.13, 1.41)	0.86 (0.80, 0.91)
*P*	7.8 × 10^-7^	2.9 × 10^-5^	1.7 × 10^-6^
BCAC IDC			
Odds ratio (95 % CI)	1.01 (0.97, 1.05)	1.05 (0.99, 1.13)	0.97 (0.93, 1.00)
*P*	0.54	0.13	0.060
Case-only			
DCIS vs IDC *P*-Het	0.0048	0.17	0.0099

*DCIS* ductal carcinoma in situ, *IDC* invasive ductal carcinoma, *BCAC* Breast Cancer Association Consortium, *ICICLE* Study to investigate the genetics of in situ carcinoma of the ductal subtype, *P*-*Het P* value for heterogeneity

Of these novel SNPs, rs12631593, 3p14.2, (an intronic variant in *FHIT*, chr3: 60726844) was the most strongly associated with DCIS (OR 1.21, 95 % CI 1.13, 1.29; *P* = 5.5 × 10^-8^). This SNP showed little association with IDC (OR 1.01, 95 % CI 0.97, 1.05; *P* = 0.54) and this was supported by the case-only analysis (*P*-Het_DCIS/IDC_ = 0.0048).

The other loci were on 22q13.2, rs73179023 (DCIS only: OR 0.85, 95 % CI 0.79, 0.90; *P* = 1.1 × 10^-6^; IDC only: OR 0.97, 95 % CI 0.93, 1.00; *P* = 0.060, *P*-Het_DCIS/IDC_ = 0.0099) and 7q21.3, rs13236351 (DCIS only: OR 1.30, 95 % CI 1.16, 1.46; *P* = 5.7 × 10^-6^; IDC only: OR 1.05, 95 % CI 0.99, 1.13; *P* = 0.13, *P*-Het_DCIS/IDC_ = 0.17).

These SNPs were genotyped in a validation study including a further 653 DCIS cases and 1,882 controls, however, for all three loci there was no evidence of an association (for rs12631593, rs13236351, and rs73179023, *P =* 0.49, 0.61, and 0.57, respectively) and none were genome wide significant following a meta-analysis of all data (*P* = 7.8 × 10^-7^, 2.9 × 10^-5^, and 1.7 × 10^-6^ respectively) (Table 3).

## Discussion

This study provides the strongest evidence to date for a shared genetic susceptibility between DCIS and IDC, based on 5,067 cases with pure DCIS (no invasive disease) and 24,670 cases with IDC. It differs from previous BCAC analyses of DCIS, as it has included an additional 3,078 DCIS cases, excluded all cases of pure LCIS and has also compared DCIS to IDC rather than all invasive disease.

An important finding of this study is the lack of DCIS/IDC-specific loci among the known breast cancer predisposition loci. Of the five breast cancer predisposition alleles originally reported by Easton et al. [24], three were shown to be associated with *in situ* (998 cases of DCIS and LCIS) disease (rs2981582-*FGFR2*, rs3803662-*TOX3*, rs889312-*MAP3K1*) with rs889312 showing a stronger association with DCIS (*P*-trend 0.007, per allele OR 1.30 for DCIS, per allele OR 1.13 for invasive disease). However, this finding of potential DCIS-specific loci was not confirmed in the Million women study which found no differential association with DCIS vs IDC for twelve breast cancer susceptibility loci, including rs889312, although their sample size was smaller (873 DCIS and 4,959 IDC) [12]. In the recent BCAC COGS analysis all 41 novel SNPs identified on the iCOGS chip had comparable ORs for invasive and *in situ* disease (based on data from 2,335 in situ, and 42,118 invasive cases), with the exceptions of rs12493607 (*TGFBR2*), and rs3903072 (11q13.1), for which associations seemed to be restricted to invasive disease [19]; however, we found no evidence of an IDC-specific association with these loci after correcting for multiple testing. A recent study investigating the association between 39 of the known breast cancer predisposition loci and breast cancer *in situ* (BCIS) suggested that rs1011970 (9p21.3, *CDKN2BAS*) had a stronger association with BCIS than invasive breast cancer (BC), *P*-Het_BCIS/BC_ = 0.0065. This trend remained in a DCIS vs BC analysis (*P*-Het_DCIS/BC_ = 0.021) [25]. Our data, however, do not support this finding (DCIS OR 1.08, 95 % CI 1.02, 1.14; *P* = 0.011; IDC OR 1.05, 95 % CI 1.0, 1.09; *P* = 0.0025, *P*-Het_DCIS/IDC_ = 0.33).

We have also shown for the first time that seven of the known invasive breast cancer predisposition loci not previously shown to be associated with DCIS have comparable ORs for IDC and DCIS: rs4973768 (*SLC4A7),* rs3821902 (*ATXN7*) [26], rs10995190 (*ZNF365*), rs554219 (*CCND1*), rs3757318 and rs2046210 (*ESR1*).

**Fig. 3:**
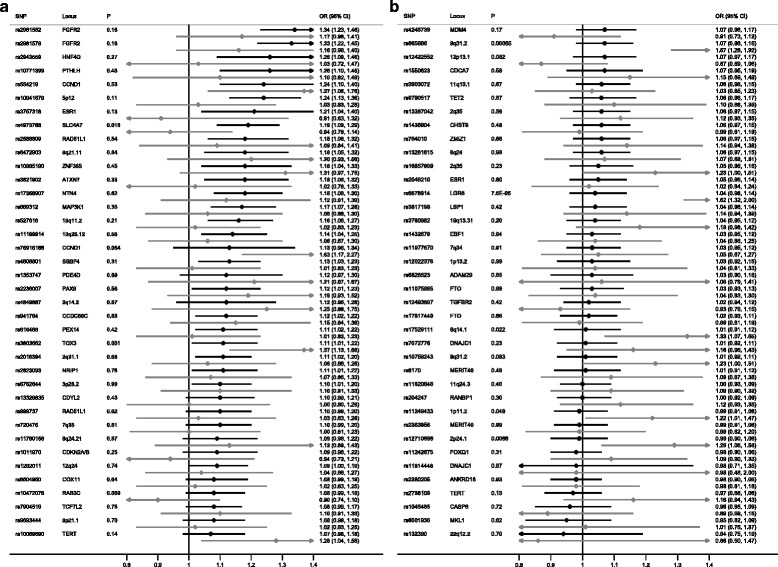
Known breast cancer predisposition loci for estrogen receptor-positive (ER+) (*black*) ductal carcinoma *in situ* and lobular carcinoma *in situ* (*gray*). Due to the large number of single nucleotide polymorphisms (SNPs), for better visual representation, the plot is split into two different sections (**a** and **b**) with a descending order of effect size for the ER+ group. *OR* odds ratio

This lack of DCIS/IDC-specific loci is in contrast to our previous study of lobular cancer in which we showed that there are loci that are specific to invasive lobular cancer (ILC), showing no association with lobular carcinoma in situ (LCIS) and there was also a suggestion of LCIS-specific loci [16]. When we compare the DCIS data presented here to our previous LCIS analyses it reveals that there is some overlap between loci that are associated with ER+ DCIS and LCIS (Fig. 3 and Additional file 14). However, there are also some differences: rs6678914, *LGR6* and rs865686, 9q31.2 are strongly associated with LCIS but there is little evidence of association with ER+ DCIS (*P*-Het_DCIS/LCIS_ = 7.4 × 10^-5^ and 6.6 × 10^-4^, respectively). We have also previously shown that rs11249433, 1p11.2 and rs11977670, 7q34 have a stronger association with invasive lobular cancer than IDC [16]. These loci were only weakly associated with LCIS and were not associated with ER+ DCIS in this analysis.

Most association studies of invasive breast cancer involve subgroup analyses based on ER status. In contrast to invasive breast cancer, ER status in DCIS is not routinely assessed in all centers despite evidence from the NSABP B-24 trial of benefit from endocrine therapy in ER+ DCIS [7]. A national audit of DCIS in the UK revealed that ER status was assessed in only 50 % of DCIS cases and ER positivity in low and intermediate grade DCIS was significantly more common than in high grade DCIS (*P* <0.001) (ER+ high grade 69 %, intermediate grade 94 %, low grade 99 %) [27]. In order to overcome this issue we performed ER immunohistochemistry on the samples from ICICLE for which ER status was unknown. However, there was still a large amount of missing data on ER status in the BCAC cases, resulting in only 684 ER– DCIS cases being available for analysis, making it difficult to draw definitive conclusions about ER– DCIS. In essence the findings are similar to invasive breast cancer, with ER– and ER+ DCIS having different genetic susceptibility profiles and ER+ DCIS having a very similar profile to ER+ IDC.

Cytonuclear grade of DCIS is used by many clinicians to select those cases most likely to benefit from radiotherapy despite the fact that grade has not been shown to be a good predictor of recurrence. In the UK audit of DCIS, grade data were available for 99 % of DCIS cases, with 59 % classified as high grade, 29 % as intermediate and 11 % as low grade [27]. Similarly, in our study data on grade were available for 95 % of cases in ICICLE. In invasive disease only a minority of predisposition loci have been shown to be grade specific; rs2981582 (*FGFR2*) and rs13281615 (8q24) [28, 29] and rs10941679 (5p12) [23]. We have shown that analysis of DCIS by grade reveals other known loci that are grade specific. The loci with the strongest association with grade were SNPs on 11q13, which had a stronger association with low/intermediate grade DCIS and IDC than high grade lesions. The finding of a strong association with low and intermediate grade ductal carcinomas that is independent of ER status in both DCIS and IDC for these loci is novel. rs614367 was the first locus on 11q13 shown to be associated with invasive breast cancer [30]. Fine mapping of the region subsequently identified two independent signals (rs554219 and rs78540526, *r*^2^ = 0.38), which are the loci reported in this analysis. Functional analyses demonstrated that the risk variants modify enhancer and silencer elements, with the likely target gene being *CCND1* [31].

A study of 150 cases of subsequent breast cancer (invasive and in situ) after DCIS observed significant association for both grade and ER status between the index DCIS and the subsequent breast cancer (whether ipsilateral or contralateral), suggesting that women with DCIS are at risk of developing subsequent breast cancers of a similar phenotype [32]. This finding supports the genetic predisposition data presented here, with ER and grade-specific loci in DCIS having similar specificity in IDC.

Although we did not identify any novel loci that reached genome wide significance, we did identify three potential novel DCIS predisposition loci, two of which were DCIS-specific (rs12631593, rs73179023), and therefore need further investigation in other cohorts of DCIS. As at least 45 % of patients with IDC have associated DCIS present at diagnosis consistent with direct precursor behavior, it may seem biologically implausible that an SNP predisposes to DCIS but is not associated with IDC. However, it is possible that there is a subset of patients with DCIS with very low probability of progression. If the finding of DCIS-specific predisposition loci were confirmed in other studies, identifying such a subset of patients with low-risk DCIS would be clinically valuable.

## Conclusion

In conclusion this is the largest study to assess genetic predisposition in DCIS and shows that the majority of invasive breast cancer predisposition loci also predispose to DCIS. It highlights that, as for invasive disease, different SNPs predispose to ER+ and ER– DCIS. In addition it shows the importance of grade in both DCIS and IDC.

## Additional files

Additional file 1:
**Study information for the Breast Cancer Association Consortium (** ***BCAC*** **) participating studies.** (DOCX 28 kb) Additional file 2:
**Sample information for the SEARCH, UKBGS, SBCS, and BBCS studies.** (DOCX 15 kb) Additional file 3:
**Number of studies and individuals included in analyses of ductal carcinoma** ***in situ*** ***(DCIS)*** **and invasive ductal carcinoma** ***(IDC)*** **.** BCAC Breast Cancer Association Consortium. (XLSX 12 kb) Additional file 4:
**Principal component analysis (PCA) results from the study to investigate the genetics of** ***in situ*** **carcinoma of the ductal subtype** ***(ICICLE)*** **.** (PPTX 142 kb) Additional file 5:
**Quantile-quantile plots from the study to investigate the genetics of in situ carcinoma of the ductal subtype (** ***ICICLE*** **).** *SNP* single nucleotide polymorphism. (PPTX 125 kb) Additional file 6:
**Grade, estrogen receptor** ***(ER)*** **status, and age groups in patients with ductal carcinoma** ***in situ*** ***(DCIS)*** **.** *BCAC* Breast Cancer Association Consortium, *ICICLE* study to investigate the genetics of ***in situ*** carcinoma of the ductal subtype. (DOCX 16 kb) Additional file 7:
**Association between ductal carcinoma** ***in situ*** ***(DCIS)*** **and known breast cancer predisposition loci.** *IDC* invasive ductal carcinoma, *P-Het P* value for heterogeneity, *SNP* single nucleotide polymorphism, *OR* odds ratio. (XLSX 19 kb) Additional file 8:
**Age-specific case-only analysis of patients with ductal carcinoma** ***in situ*** **(DCIS) diagnosed at age <50 vs ≥50 years.** *P-Het P* value for heterogeneity, *SNP* single nucleotide polymorphism, *OR* odds ratio. (XLSX 17 kb) Additional file 9:
**Associations between the known breast cancer predisposition loci and estrogen receptor-positive** ***(ER+)*** **or ER– ductal carcinoma** ***in situ*** ***(DCIS)*** **.** *P-Het P* value for heterogeneity, *SNP* single nucleotide polymorphism, *OR* odds ratio. (XLSX 19 kb) Additional file 10:
**a, b Known breast cancer predisposition loci for low/intermediate grade** ***(black)*** **and high grade ductal carcinoma** ***in situ*** **(DCIS)** ***(gray)*** **.** Due to the large number of single nucleotide polymorphisms (*SNP*s), the plot is split for better visual representation into two different sections (a and b) with a descending order of effect size for the low/intermediate group. *OR* odds ratio. (ZIP 20 kb) Additional file 11:
**Associations of the known breast cancer predisposition loci for high and low-intermediate grade ductal carcinoma** ***in situ*** ***(DCIS)*** **.** *P-Het P* value for heterogeneity, *OR* odds ratio. (XLSX 18 kb) Additional file 12:
**Association of rs75915166 and rs554219 with grade in invasive ductal carcinoma (** ***IDC*** **).** *P-Het P* value for heterogeneity, *OR* odds ratio. (XLSX 9 kb) Additional file 13:
**Associations between the known and novel breast cancer predisposition loci and invasive ductal cancer, by estrogen receptor (** ***ER*** **) status and grade.** *OR* odds ratio. (XLSX 24 kb) Additional file 14:
**Association between the known breast cancer predisposition loci and estrogen receptor-positive** ***(ER+)*** **ductal carcinoma** ***in situ*** ***(DCIS)*** **or lobular carcinoma** ***in situ*** ***(LCIS)*** **.** *P-Het P* value for heterogeneity, *SNP* single nucleotide polymorphism, *OR* odds ratio. (XLSX 19 kb)

## Abbreviations

ABCS: Amsterdam Breast Cancer Study
BBBC: Bavarian Breast Cancer Cases and Controls
BBCS: British Breast Cancer Study
BC: breast cancer
BCAC: Breast Cancer Association Consortium
BCIS: breast carcinoma in situ
CI: confidence interval
COGS: Collaborative Oncological Gene-Environment Study
DCIS: ductal carcinoma in situ
ER: Estrogen receptor
H&E: hematoxylin and eosin
HWE: Hardy Weinberg equilibrium
ICICLE: study to investigate the genetics of in situ carcinoma of the ductal subtype
IDC: invasive ductal carcinoma
LCIS: lobular carcinoma in situ
MAF: minor allele frequency
OR: odds ratio
PCA: principal component analysis
*P*-*Het*: *P* value for heterogeneity
SNP: single nucleotide polymorphism
