# Supplementary material for: Genetic predisposition to ductal carcinoma in situ of the breast
Source: Breast Cancer Res. 2016 Feb 17;18:22. doi: 10.1186/s13058-016-0675-7 (PMC4756509; doi:10.1186/s13058-016-0675-7)
Supplement: Additional file 1: — Study information for the Breast Cancer Association Consortium ( BCAC ) participating studies. (DOCX 28 kb) [file 13058_2016_675_MOESM1_ESM.docx]

**Additional File 1. Study information for BCAC participating studies**

| **Study Acronym** | **Study Name** | **Country** | **Recruitment base**  **Cases Controls** | |
| --- | --- | --- | --- | --- |
| ABCS | Amsterdam Breast Cancer Study | Netherlands | (ABCS-F) All non-BRCA1/2 breast cancer cases from the family cancer clinic of the NKI-AVL tested in the period 1995-2009; all ages and diagnosed with breast cancer in 1965-2008.. | Randomly selected women from population-based prospective cohort studies, aged <50 at baseline (1987-1991 and 1993-1997) and from the same areas as cases. |
| BBCC | Bavarian Breast Cancer Cases and Controls | Germany | Consecutive, unselected cases with invasive breast cancer recruited at the University Breast Centre, Franconia in Northern Bavaria from 2002-2006 | Healthy women aged 55 or older with no diagnosis of cancer. Invited by a newspaper advertisement in Northern Bavaria between 2002-2006 |
| BBCS | British Breast Cancer Study | U.K. | (i) English & Scottish Cancer Registries: all breast cancer cases who developed a first primary before age 66 in 1971 or later and who subsequently developed a second primary cancer (ii) Breast Cancer Clinics: all breast cancer cases who developed a first primary before age 71 in 1967 or later and who either subsequently developed a second primary or had at least two affected female first-degree relatives.  All recruited from 2001-2008. | A friend, sister-in-law, daughter-in-law or other non-blood relative of cases, recruited from 2001-2008 |

| BIGGS | Breast Cancer in Galway Genetic Study | Ireland | Unselected cases recruited from University College Hospital Galway and surrounding hospitals in the West of Ireland since 2001 | Women > 60 years with no personal history of any cancer and no family history of breast or ovarian cancer identified from retirement groups in the West of Ireland between 2001-2008. |
| --- | --- | --- | --- | --- |
| BSUCH | Breast Cancer Study of the University Clinic Heidelberg | Germany | All cases diagnosed with breast cancer in 2007-2009 at the University Women`s Clinic Heidelberg | Female blood donors recruited in 2007- 2009 at the Institute of Transfusion Medicine & Immunology, Mannheim. |
| CECILE | CECILE Breast cancer study | France | All cases diagnosed with breast cancer in 2005-2007 among women <75 years of age residing in the *départements* of Ille-et-Vilaine and Côte d'Or . Cases were recruited from the main cancer treatment center (Centre Eugène-Marquis in Rennes and Centre Georges-François-Leclerc in Dijon) and from other private or public hospitals in each area. | General population control women residing in the same areas as the cases (Ille-et-Vilaine and Côte d’Or). Controls were frequency-matched to the cases by 5-year age groups. They were recruited in 2005-2007 using a random digit dialing procedure and quotas by socioeconomic status to reflect the distribution by SES of the population in each area. |
| CGPS | Copenhagen General Population Study | Denmark | Consecutive, incident cases from one hospital with centralized care for a population of 400,000 women in Copenhagen (2001-present) | Women with no history of breast cancer residing in the same region as cases identified from the Copenhagen General Population Study (2003-2007) |
| CNIO-BCS | Spanish National Cancer Centre Breast Cancer Study | Spain | (i) consecutive breast cancer patients from three public hospitals, two in Madrid and one in Oviedo;  (ii) cases with at least one affected first degree relative recruited through the CNIO family cancer clinic in Madrid (2000-2005) | Women attending the Menopause Research Centre, Madrid and female members of the College of Lawyers attending a free, targeted medical check-up in Madrid, all free of breast cancer and all in Madrid between 2000-2005 |
| ESTHER | ESTHER Breast Cancer Study | Germany | Breast cancer cases in all hospitals in the state of Saarland, from 2001-2003 (ESTHER) and 1996-1998 (VERDI) | Random sample of women at routine health check-up in Saarland, in 2000-2002; frequency matched to cases by age in-5 year categories |
| GC-HBOC | German Familial Breast Cancer Study | Germany | Index patients from German breast cancer families; BRCA1/2 mutation free, collected 1996-2007 via Institute of Human Genetics, University Heidelberg Department of Gynaecology & Obstetrics, Cologne & Department of Gynaecology and Obstetrics at the Ludwig-Maximilians-University, Munich | Female blood donors recruited in 2004 & 2007 at the Institute of Transfusion Medicine & Immunology, Mannheim. |
| HEBCS | Helsinki Breast Cancer Study | Finland | (1) Consecutive cases (883) from the Department of Oncology, Helsinki University Central Hospital 1997-8 and 2000, (2) Consecutive cases (986) from the Department of Surgery, Helsinki University Central Hospital 2001 – 2004, (3) Familial breast cancer patients (536) from the Helsinki University Central Hospital, Departments of Oncology and Clinical Genetics (1995-) | Healthy females from the same geographical region in Southern Finland in 2003. |
| HMBCS | Hannover-Minsk Breast Cancer Study | Belarus | Cases from the Byelorussian Institute for Oncology and Medical Radiology Aleksandrov N.N. in Minsk or at one of 5 regional oncology centers in Gomel, Mogilev, Grodno, Brest or Vitebsk (2002-2008) | Women attending general medical examination at gynecology clinics in Gomel, Mogilev, Grodno, Brest or Vitebsk; women attending the Institute for Inherited Diseases in Minsk; female blood donors in Minsk; healthy relatives of cases (2002-2008) |

| KBCP | Kuopio Breast Cancer Project | Finland | Women seen at Kuopio University Hospital between 1990-1995 because of a breast lump, mammographic abnormality, or other breast symptom and who were found to have breast cancer | Selected from the National Population Register between 1990-1995; age and long-term area-of-residence matched to cases |
| --- | --- | --- | --- | --- |
| kConFab/  AOCS | Kathleen Cuningham Foundation Consortium for Research into Familial Breast Cancer / Australian Ovarian Cancer Study | Australia | Index (youngest affected) cases from *BRCA1*- and *BRCA2*-mutation-negative multiple-case breast and breast-ovarian families recruited though family cancer clinics from across Australia and New Zealand from 1998-present | Identified from the electoral rolls from across Australia as part of the Australian Ovarian Cancer Study in 2002-2006 |
| LMBC | Leuven Multidisciplinary Breast Centre | Belgium | All patients diagnosed with breast cancer and seen in the Multidisciplinary Breast Center in Leuven (Gashuisberg) since June 2007 plus retrospective collection of cases diagnosed since 2000 | Blood donors at Gasthuisberg Hospital (200-2008) |
| MARIE | Mammary Carcinoma Risk Factor Investigation | Germany | Incident and prevalent cases diagnosed from 2001-2005 in the study region Hamburg in Northern Germany, and from 2002-2005 in the study region Rhein-Neckar-Karlsruhe in Southern Germany. | 2 controls per case were randomly drawn from population registries and frequency matched by birth year and study region to the case. Controls were recruited from 2002 to 2006. |
| MBCSG | Milan Breast Cancer Study Group | Italy | Familial and/or early onset breast cancer patients (aged 22-87) negative for mutations in *BRCA1* and *BRCA2*, ascertained at two large cancer centers in Milan from 2000-present | Female blood donors recruited at two centres in Milan from 2004-present and 2007-present |
| MCBCS | Mayo Clinic Breast  Cancer Study | U.S.A. | Incident cases residing in 6 states (MN, WI, IA, IL,  ND, SD) seen at the Mayo Clinic in Rochester, MN  from 2002-2010. | Women presenting for general  medical examination at the Mayo  Clinic from 2002-2010; frequency  matched to cases on age, ethnicity  and county/state. |

| MEC | Multiethnic Cohort | USA | Incident cases identified from SEER cancer registries in Los Angeles County & State registries in California & Hawaii, USA from 1993-2002. Grouped by self-reported ethnicity. | Women without cancer from the same States, recruited concurrently with cases & frequency matched to cases by age at blood-draw & self- reported ethnicity |
| --- | --- | --- | --- | --- |
| OBCS | Oulu Breast Cancer Study | Finland | Consecutive incident cases diagnosed at the Oulu University Hospital between 2000-2004 | Female blood donors recruited in 2002 from the same geographical region in Northern Finland |
| OFBCR | Ontario Familial Breast Cancer Registry | Canada | Invasive cases aged 20-54 years  identified from the Ontario Cancer Registry from 1996-1998. All those at high genetic risk were eligible; random samples of women not meeting these criteria were also asked to participate. During 2001-2005, enrollment was limited to minority and high-risk families. | Identified by calling randomly selected residential telephone numbers in the same geographical region from 1998-2001; frequency matched to cases by age in 5 year categories |
| ORIGO | Leiden University Medical Centre Breast Cancer Study | Netherlands | Consecutive case patients diagnosed 1996–2006 in 2 hospitals in South–West Netherlands (Leiden & Rotterdam). No selection for family history; Rotterdam case patients selected for diagnosis aged <70. Case patients with in situ carcinomas eligible. | (1) Blood bank healthy donors from Southwest Netherlands recruited in 1996, 2000 or 2007; (2) People who married a person who was part of a family with high breast cancer risk (BRCA1/2/x). From the Southwest of the Netherlands, 1990–1996; (3) Females tested at the local clinical genetics department for familial diseases, excluding familial cancer syndromes (no mutation found in gene(s) related to the disease being tested), 1995–07 |
| pKARMA | Karolinska  Mammography Project for Risk Prediction of  Breast Cancer – prevalent cases | Sweden | Incident cases from Jan 2001 – Dec 2008 from the  Stockholm/Gotland area. Identified through the  Stockholm breast cancer registry. | Unmatched participants of the  KARMA mammography screening  study recruited between 2010 and  2011 from Helsingborg and  Stockholm. |
| RBCS | Rotterdam Breast Cancer Study | Netherlands | Familial breast cancer patients selected from the clinical genetics center at Erasmus Medical Center between 1994-2005 | Spouses or mutation-negative siblings of heterozygous Cystic Fibrosis mutation carriers selected from the clinical genetics centre at Erasmus Medical Center between 1996-2006 |
| RPCI | Roswell Park Cancer  Institute | U.S.A | Triple negative invasive breast cancer cases from  incident cases recruited to the RPCI Data Bank  and Biorepository. | Healthy controls identified from  employee volunteers, and women  recruited from community events. |
| SBCS | Sheffield Breast Cancer Study | U.K. | Women with breast cancer recruited in 1998-2005 at surgical outpatient clinics at the Royal Hallamshire Hospital, Sheffield | Unselected women attending the Sheffield Mammography Screening Service in 2000-2004 with no evidence of a breast lesion |
| SEARCH | Study of Epidemiology & Risk Factors in Cancer Heredity | U.K. | Identified through the East Anglian Cancer Registry: (i) 1991-1996: alive, prevalent cases diagnosed before age 55; (ii) since 1996: incident cases diagnosed before age 70 diagnosed after 1996 | (a) Women from the same geographic region selected from the EPIC-Norfolk cohort study, 1992-1994 (b) women attending GP practices, frequency matched to cases by age and geographic region (2003-present) |
| SKKDKFZS* | Städtisches Klinikum Karlsruhe Deutsches Krebsforschungszentrum Study |  | Women diagnosed with primary *in situ* or invasive breast cancer at the Städtisches Klinikum Karlsruhe from March 1993 to July 2005. Cases were 21-93 years of age. | Controls for triple negative cases were from an unselected series of unaffected women from the same geographical region |
| SZBCS | IHCC-Szczecin Breast Cancer Study | Poland | Prospectively ascertained cases of invasive breast cancer patients diagnosed at the Regional Oncology Hospital (2002-2003 and 2006-2007) or the University Hospital (2002-2007), both in Szczecin, West Pomerania, Poland. | Selected from a population-based study of the 1.3 million inhabitants of West Pomerania (2003-2004); matched to cases for year of birth, sex and region |
| UKBGS | Breakthrough Generations Study | UK | Cohort members who developed breast cancer or in situ breast cancer after entry into the Breakthrough Generations Study (cohort of >100,000 women followed up for breast cancer, recruited from the UK during 2003-2010). | Women who had not had breast cancer or in situ breast cancer selected 1:1 matching to cases on date of birth, year of entry in to the study (2003-2010), source of recruitment, availability of blood sample and ethnicity. |

^*^SKKDKFZS belongs to the BCAC but was genotyped as part of the triple negative consortium (TNBCC).
