# Supplementary material for: Genetic predisposition to ductal carcinoma in situ of the breast
Source: Breast Cancer Res. 2016 Feb 17;18:22. doi: 10.1186/s13058-016-0675-7 (PMC4756509; doi:10.1186/s13058-016-0675-7)
Supplement: Additional file 2: — Sample information for the SEARCH, UKBGS, SBCS, and BBCS studies. (DOCX 15 kb) [file 13058_2016_675_MOESM2_ESM.docx]

**Additional File 2. Sample information for SEARCH, UKBGS, SBCS, and BBCS studies**

| **UK study** | **N^o^ of DCIS**  **cases** | **Total N^o^ of controls** | **Source of controls** | **N^o^ of controls selected for BCAC analysis** | **N^o^ of controls selected for ICICLE analysis** |
| --- | --- | --- | --- | --- | --- |
| **BBCS**  British Breast Cancer Study | 108 | 1,397 | A friend or non-blood relative of cases, recruited from throughout UK | 166 | 1,231 |
| **SBCS**  Sheffield Breast Cancer Study | 61 | 848 | Unselected women attending Sheffield Mammography Screening Service with no evidence of a breast lesion | 144 | 704 |
| **UKBGS**  Breakthrough Generations Study | 60 | 470 | Women from throughout the UK who had not had breast cancer or in situ disease before entry into the cohort study | 100 | 370 |
| **SEARCH**  Study of Epidemiology & Risk Factors in Cancer Heredity | 171 | 8,069 | (a) from the EPIC-Norfolk cohort study, (b) women attending GP practices, matched to cases by age and geographic region (East Anglia) | 5,374 | 2,695 |
| **Total** | **400** | **10,784** |  | **5,784** | **5,000** |
