# Supplementary material for: Genetic predisposition to ductal carcinoma in situ of the breast
Source: Breast Cancer Res. 2016 Feb 17;18:22. doi: 10.1186/s13058-016-0675-7 (PMC4756509; doi:10.1186/s13058-016-0675-7)
Supplement: Additional file 4: — Principal component analysis (PCA) results from the study to investigate the genetics of in situ carcinoma of the ductal subtype (ICICLE) . (PPTX 142 kb) [file 13058_2016_675_MOESM4_ESM.pptx]

## Slide 1
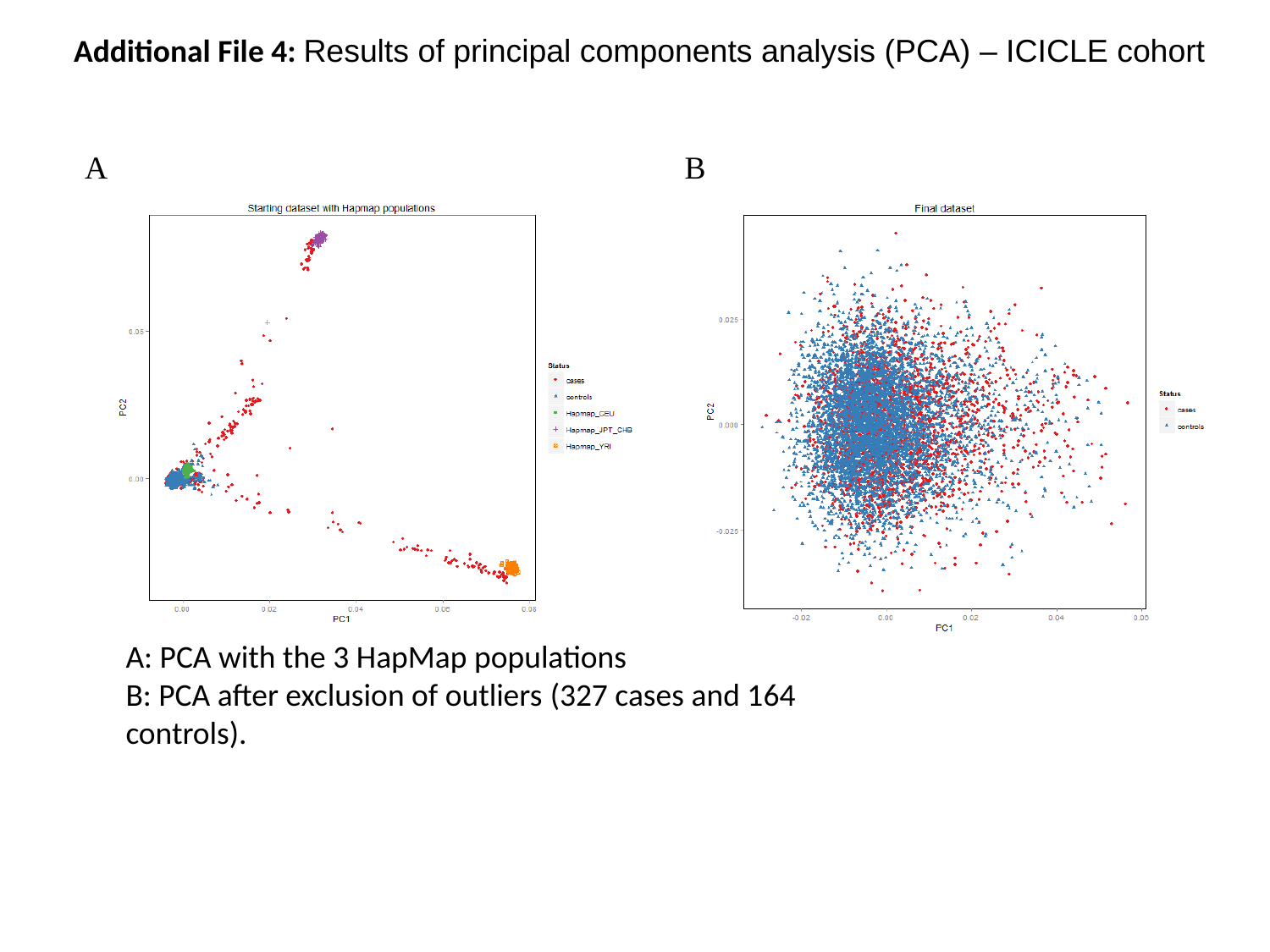

Additional File 4: Results of principal components analysis (PCA) – ICICLE cohort
A
B
A: PCA with the 3 HapMap populations
B: PCA after exclusion of outliers (327 cases and 164 controls).
