# Supplementary material for: Genetic predisposition to ductal carcinoma in situ of the breast
Source: Breast Cancer Res. 2016 Feb 17;18:22. doi: 10.1186/s13058-016-0675-7 (PMC4756509; doi:10.1186/s13058-016-0675-7)
Supplement: Additional file 5: — Quantile-quantile plots from the study to investigate the genetics of in situ carcinoma of the ductal subtype ( ICICLE ). SNP single nucleotide polymorphism. (PPTX 125 kb) [file 13058_2016_675_MOESM5_ESM.pptx]

## Slide 1
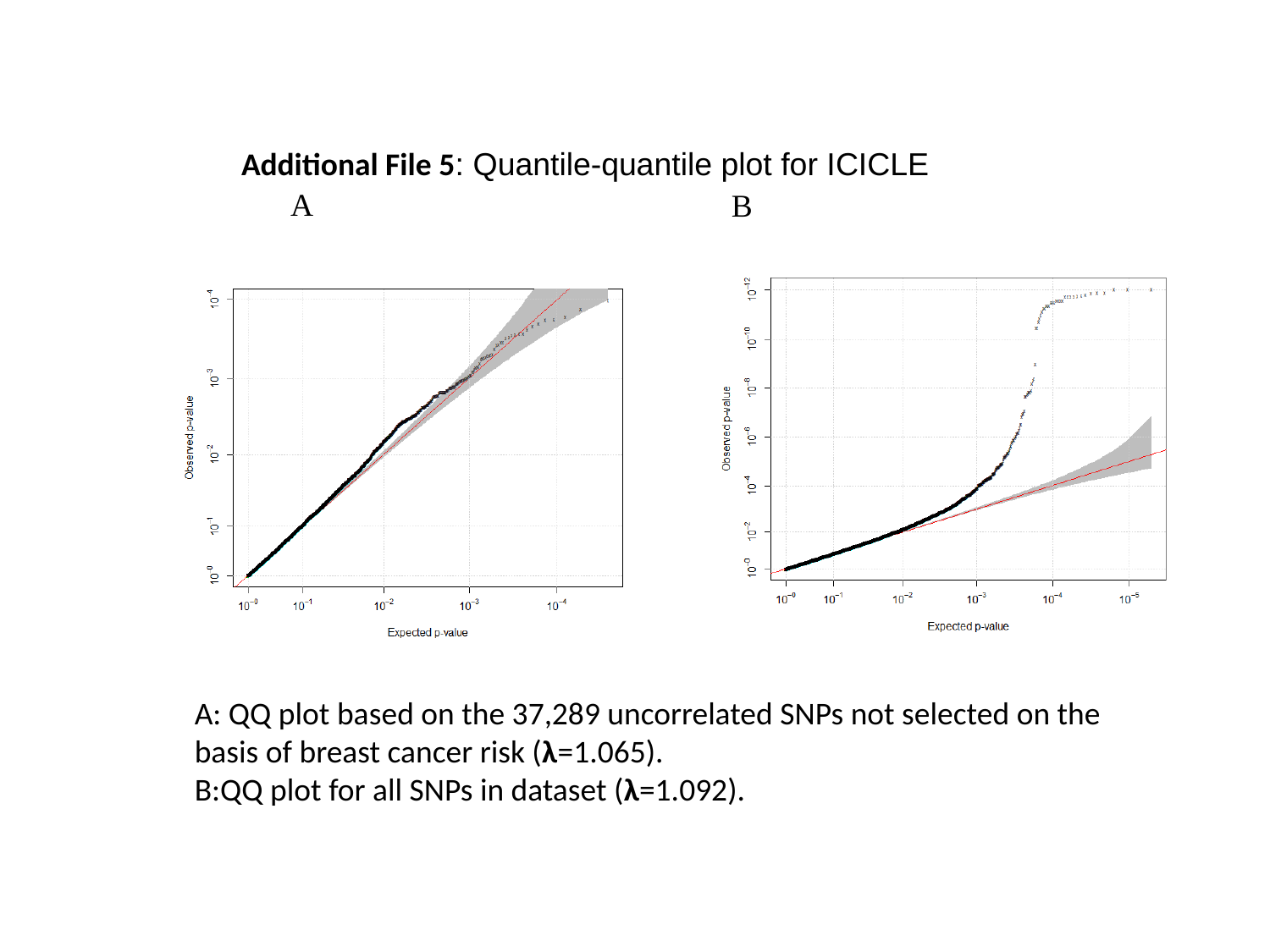

Additional File 5: Quantile-quantile plot for ICICLE
A
B
A: QQ plot based on the 37,289 uncorrelated SNPs not selected on the basis of breast cancer risk (λ=1.065).
B:QQ plot for all SNPs in dataset (λ=1.092).
