# Supplementary material for: Genetic predisposition to ductal carcinoma in situ of the breast
Source: Breast Cancer Res. 2016 Feb 17;18:22. doi: 10.1186/s13058-016-0675-7 (PMC4756509; doi:10.1186/s13058-016-0675-7)
Supplement: Additional file 6: — Grade, estrogen receptor (ER) status, and age groups in patients with ductal carcinoma in situ (DCIS) . BCAC Breast Cancer Association Consortium, ICICLE study to investigate the genetics of in situ carcinoma of the ductal subtype. (DOCX 16 kb) [file 13058_2016_675_MOESM6_ESM.docx]

**Additional File 6a**: Grade status in DCIS cases

| **Grade** | **N^o^ of ICICLE cases** | **N^o^ of BCAC cases** |
| --- | --- | --- |
| High | 1635 | 306 |
| Intermediate | 693 | 247 |
| Low | 250 | 275 |
| Missing | 137 | 1524 |
| **Total** | **2715** | **2352** |

**Additional File 6b**: ER status in DCIS cases

| **ER status** | **N^o^ of ICICLE cases** | **N^o^ of BCAC cases** |
| --- | --- | --- |
| Positive | 1484 | 664 |
| Negative | 383 | 301 |
| Missing | 848 | 1387 |
| **Total** | **2715** | **2352** |

**Additional File 6c**: Age of diagnosis in DCIS cases

| **Age of diagnosis** | **N^o^ of ICICLE cases** | **N^o^ of BCAC cases** |
| --- | --- | --- |
| <50 | 573 | 410 |
| ≥50 | 2003 | 1648 |
| Missing | 139 | 294 |
| **Total** | **2715** | **2352** |
