# Supplementary figures and images for: Genetic predisposition to ductal carcinoma in situ of the breast
Source: Breast Cancer Res. 2016 Feb 17;18:22. doi: 10.1186/s13058-016-0675-7 (PMC4756509; doi:10.1186/s13058-016-0675-7)

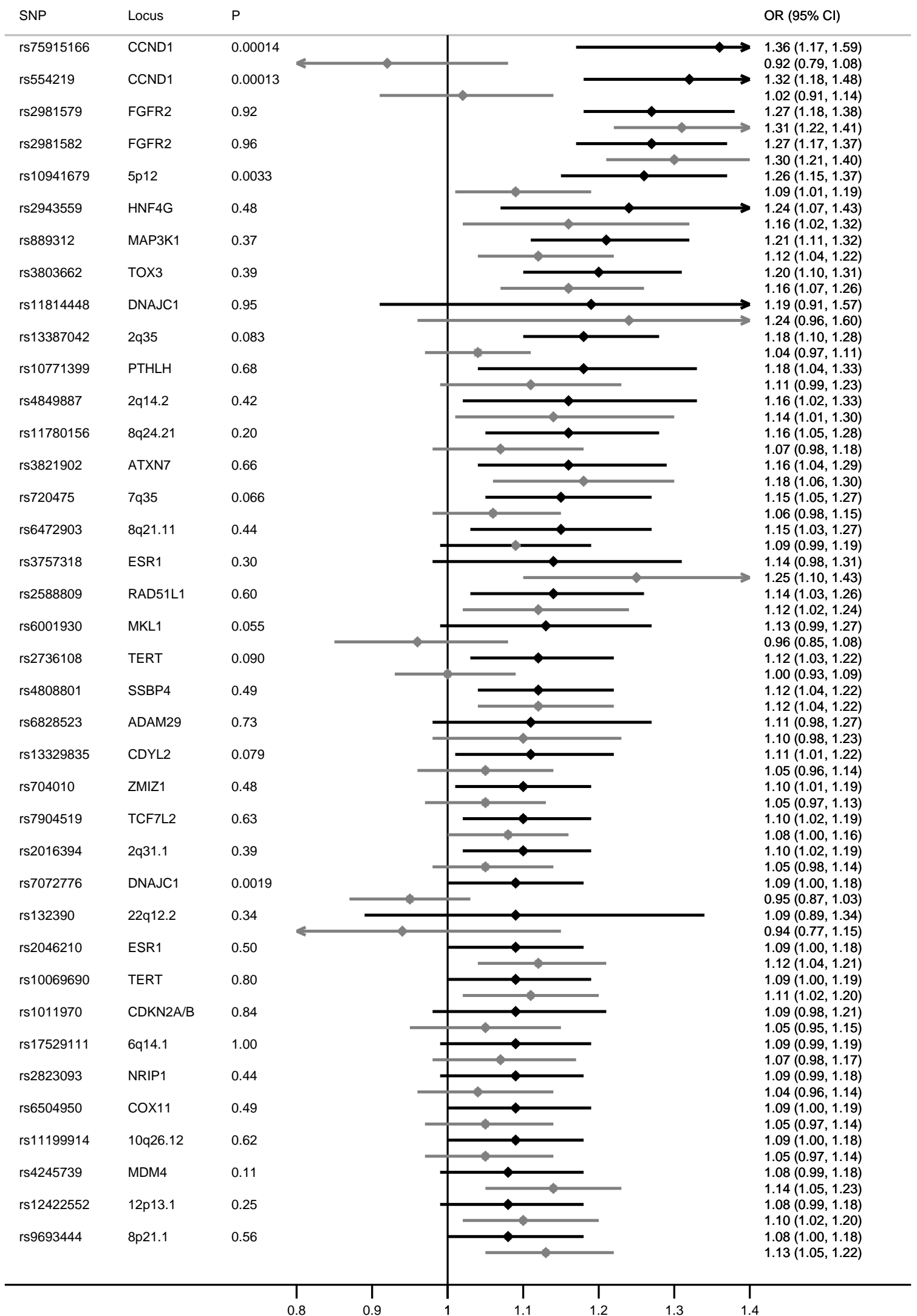

Supplement: Additional file 10: — a, b Known breast cancer predisposition loci for low/intermediate grade (black) and high grade ductal carcinoma in situ (DCIS) (gray) . Due to the large number of single nucleotide polymorphisms (SNPs), the plot is split for better visual representation into two different sections (a and b) with a descending order of effect size for the low/intermediate group. OR odds ratio. (ZIP 20 kb) [file 13058_2016_675_MOESM10_ESM.zip › Additional File 10/Additional File 10A.pdf]

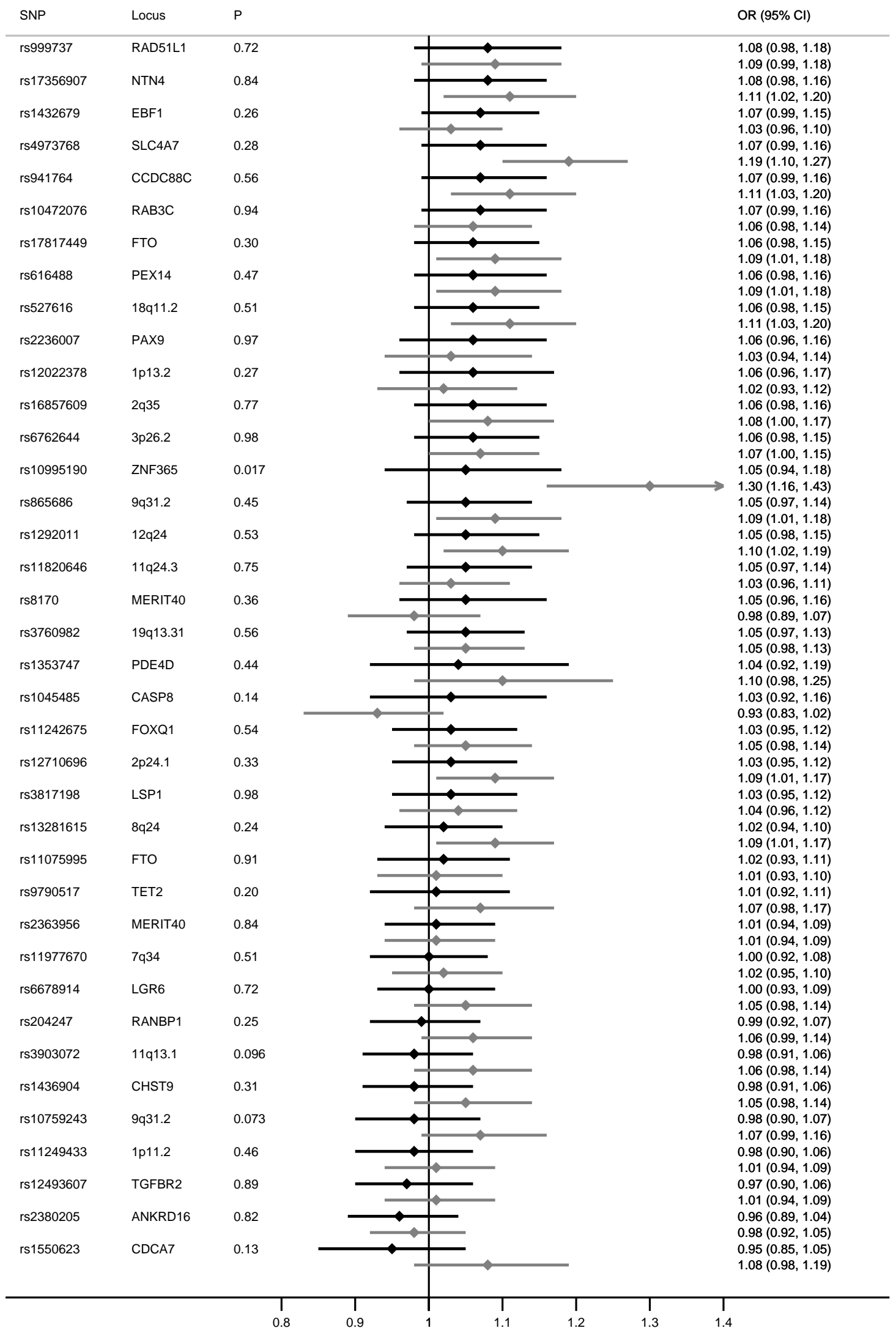

Supplement: Additional file 10: — a, b Known breast cancer predisposition loci for low/intermediate grade (black) and high grade ductal carcinoma in situ (DCIS) (gray) . Due to the large number of single nucleotide polymorphisms (SNPs), the plot is split for better visual representation into two different sections (a and b) with a descending order of effect size for the low/intermediate group. OR odds ratio. (ZIP 20 kb) [file 13058_2016_675_MOESM10_ESM.zip › Additional File 10/Additional File 10B.pdf]
